# The relationship between adolescents’ self-regulated learning and academic achievement: an interrelated mediation model of academic emotions: evidence from a nationwide sample in China

**DOI:** 10.3389/fpsyg.2026.1768675

**Published:** 2026-02-24

**Authors:** Rui Sun, Sheng Zhang

**Affiliations:** Collaborative Innovation Center of Assessment toward Basic Education Quality, Beijing Normal University, Beijing, China

**Keywords:** a large survey, academic achievement, academic emotions, self-regulated learning, teacher-student relationships

## Abstract

**Introduction:**

In the 21st century, self-regulated learning (SRL) plays a vital role in the cultivation of high-quality talent. Grounded in self-regulated learning theory and attachment theory, this study aims to systematically examine the relationship between adolescents’ SRL and academic achievement, considering the roles of academic emotions and teacher–student relationships (TSR). Additionally, the study investigates whether a recursive pathway exists from academic emotions back to SRL.

**Methods:**

The study draws on nationwide survey data from China (*N* = 88,149 students) and employs structural equation modeling (SEM) to analyze the relationships among SRL, academic emotions, TSR, and academic achievement. Academic emotions were included as a mediating variable, and TSR as a moderating variable.

**Results:**

The results indicate that: (1) adolescents’ levels of SRL are significantly and positively associated with their academic achievement; (2) three types of academic emotions (e.g., positive high-arousal emotions) significantly mediate the relationship between SRL and academic achievement; and (3) TSR moderate both the first stage of the mediation pathway (“SRL → academic emotions → academic achievement”) and the direct effect of SRL on academic achievement.

**Discussion:**

Overall, the findings support the cognition–emotion cyclical model, suggesting that students can enhance SRL through positive academic emotions, while appropriate SRL strategies can in turn foster positive academic emotions, forming a virtuous cycle that strengthens students’ autonomy in the learning process. This study extends Pintrich’s SRL model by empirically validating a recursive loop between SRL and academic emotions. Furthermore, it highlights a “dual empowerment” path of emotional regulation and metacognitive monitoring through optimized TSR, offering vital policy insights for fostering high-order autonomous learning in the AI era.

## 1 Introduction

In today’s rapidly evolving knowledge economy, advanced technologies are driving the intelligent transformation of traditional educational models. Although the rapid development of technology provides unprecedented convenience for students’ self-regulated learning, excessive reliance on technology may undermine students’ ability to learn independently. On one hand, in an educational context increasingly integrated with artificial intelligence, strengthening students’ self-regulated learning capabilities is essential for implementing student-centered education; on the other hand, self-regulated learning serves as a key tool for addressing adolescents’ learning challenges and promoting high-quality learning (Sun and Chen, 2024). Research indicates that adolescents can improve their learning initiative, promote all-round development, and adapt more effectively to a rapidly changing society through self-regulated learning (Feng et al., 2025). In this process, self-regulated learning plays a critical role. Therefore, a more profound understanding of the underlying psychological mechanisms of adolescents’ SRL, along with effective environmental and psychological interventions, is crucial for addressing the challenges posed by technological advancements.

In recent years, academic emotions and teacher-student relationships (TSR) have been increasingly recognized as critical factors related to SRL. Although many studies have demonstrated that academic emotions significantly influence students’ motivation, learning strategies, and academic outcomes, most studies focus solely on the path from academic emotions to SRL (e.g., Webster and Hadwin, 2015; Pekrun et al., 2002; Pekrun, 2006). Few studies have analyzed the potential pathway connecting SRL and academic emotions and the underlying mechanisms, considering possible reciprocal relationships between the two. Furthermore, TSR, as a crucial source of environmental support, play an important part in moderating students’ psychological and behavioral responses to learning. However, it remains unclear whether empirical evidence from large-scale data supports the hypothesized upward cycle between SRL and academic emotions (Feraco et al., 2023). How do cognition (SRL), emotion, and social relationships interact with one another? These questions warrant further research, which could provide theoretical support and practical insights for improving students’ learning abilities and adapting the education system to the changes of AI-integrated educational landscape.

## 2 Literature review

### 2.1 SRL and academic performance

According to Self-Determination Theory (SDT; Deci and Ryan, 2008) and Self-Regulated Learning theory (SRLT, Pintrich, 2000), SRL refers to students’ ability to independently select, integrate, and apply metacognitive, cognitive, and motivational-affective strategies. It encompasses skills such as organizing and transforming information, self-reinforcement, seeking relevant information, rehearsing, and using mnemonic techniques. Students with strong SRL skills are able to make clear value judgments and cognitions about their learning and its processes. As a result, they can effectively guide their own learning, which in turn translates into enhanced academic skills (Kaplan, 2008). Numerous studies have confirmed that SRL is positively associated with academic performance (Broadbent and Poon, 2015; Cleary and Platten, 2013; Mega et al., 2014). Both metacognitive and cognitive strategies contribute to achievement, with metacognition appearing to have a relatively stronger link (Chiu et al., 2007; Ghiasvand, 2010). Moreover, SRL appears to be linked to academic achievement both directly and indirectly through mediators such as self-control and learning engagement (Cleary and Zimmerman, 2012). Building on the theoretical and empirical research presented above, this study proposes the following hypotheses: Hypothesis 1: SRL is positively associated with students’ academic achievement (H1).

### 2.2 The mediating effect of academic emotions

Academic emotions refer to the various emotional experiences students encounter during learning processes that are related to their academic activities. These emotions include enjoyment, boredom, disappointment, anxiety, and anger (Pekrun, 2006). In previous studies, the correlation between students’ learning behaviors and academic emotions has often been conceptualized within a dual-process framework (Boekaerts, 2011). First, academic emotions are conceptualized as motivational forces that activate or drive behavior, a perspective grounded in the theoretical framework of positive psychology (Seligman and Csikszentmihalyi, 2014). Second, SD theory emphasizes that SRL promotes students’ self-regulation through a recursive cycle consisting of three stages: motivation, action, and self-reflection (Feraco et al., 2023). Base on the Control Value Theory (Pekrun, 2006), adolescents’ perceptions of control over study tasks and the value they assign to these tasks are important antecedents of academic emotions. Previous research has revealed that students with higher perceived self-regulated control, intrinsic value, and attainment value attainment value are more likely to experience positive academic emotions (Forsblom et al., 2021; Putwain et al., 2018). Moreover, empirical studies have revealed that students’ feelings of boredom were negatively correlated with their perceived self-regulation levels (Forsblom et al., 2021; Pekrun et al., 2010), which aligns with theoretical framework of the Control-Value Theory (CVT).

Meanwhile, numerous studies have shown that academic emotions impact achievement. For example, test anxiety reduces working memory, negatively impacting performance (Seipp, 1991). All four types of academic emotions significantly affect academic achievement: positive emotions improve performance, while negative emotions hinder it (Pekrun et al., 2017; Tan et al., 2021). Although research supports academic emotions as mediators between SRL and performance, research on how SRL affects academic emotions remains limited. Whether students’ level of SRL can enhance positive academic emotions and thereby improve academic performance requires further empirical verification. Therefore, this study proposes the following hypotheses:

- *H2.1*: Positive high-arousal emotions positively mediate the relationship between SRL and academic achievement.
- *H2.2*: Positive low-arousal emotions positively mediate the relationship between SRL and academic achievement.
- *H2.3*: Negative high-arousal emotions negatively mediate the relationship between SRL and academic achievement.
- *H2.4*: Negative low-arousal emotions negatively mediate the relationship between SRL and academic achievement.

### 2.3 The moderating role of positive TSR

TSR is the cognitive, emotional, and behavioral bond formed through daily instructional and interpersonal interactions, classified as avoidant, secure, or ambivalent (Pianta et al., 1995). According to attachment theory and SDT (Ainsworth et al., 2015), students have an inherent need for connections with others, such as teachers (relatedness). A positive TSR facilitates a more relaxed and enjoyable learning experience, and fosters stronger positive academic emotions in students. Research has revealed that individuals can maintain higher levels of intrinsic motivation or regulate learning engagement through positive TSR. Specifically, close TSR appears to be positively related to students’ flexibility in the use of study strategies, and the effective application of these strategies, in turn, improves academic achievement (e.g., Sainio et al., 2023; Tan et al., 2024). Moreover, supportive TSR can encourage teachers to adopt more student-centered instructional approaches and actively enhance students’ intrinsic motivation and learning engagement, thereby promoting academic achievement (e.g., Roorda et al., 2017). Moreover, longitudinal study show that teacher support positively impacts students’ academic achievement throughout adolescence (Košir and Tement, 2014). However, TSR is not always associated with higher academic outcomes. For instance, negative TSR hinders the fulfillment of students’ relevant needs and may weaken their learning performance (e.g., Hajovsky et al., 2017). Despite these results, the moderating role of TSR in the interplay among SRL, academic emotions, and academic achievement remains unclear. Therefore, further exploration is warranted in the present study. Given this, the study formulates the hypothesis that: Hypothesis 3: positive TSR moderates both the direct effect of SRL on academic achievement and the mediating effect of academic emotions (H3).

**Figure 1:**
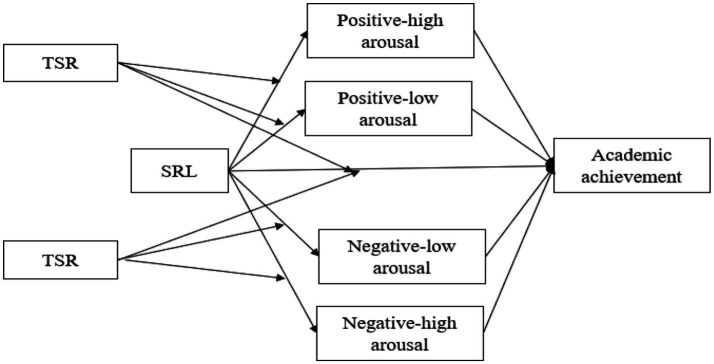
The path coefficients of moderated mediation model.

In summary, existing research on this topic mainly focuses on the relationships among academic emotions, SRL, and academic achievement, often examining academic emotions as the independent variable and SRL as the mediating variable. However, within the framework of SDT, the recursive and cyclical interaction between academic emotions and SRL has rarely been examined empirically. Previous studies have also not clearly articulated the relationships between and SRL level and academic achievement, particularly regarding their potential reciprocal associations with academic emotions. In addition, the moderating role of different levels of positive TSR has received insufficient empirical attention. Therefore, the current study draws on the SRL model and SDT to explain the relationships among these variables. The study aims to develop a moderated mediation model (see Figure 1) to further investigate the relationships and underlying mechanisms among SRL, positive high-arousal emotions, positive low-arousal emotions, negative high-arousal emotions, negative low-arousal emotions, and academic achievement. Based on the model’s findings, the study also seeks to provide practical recommendations for enhancing SRL in adolescents.

## 3 Materials and methods

### 3.1 Participants

The data used in this study were provided by the Collaborative Innovation Center of Assessment for Basic Education Quality at Beijing Normal University. A multi-stage stratified sampling design combined with probability proportional to size (PPS) sampling was employed to recruit primary and secondary school students across 31 provincial-level administrative regions in China. First, counties within each province were stratified according to per capita GDP. Within each selected county, schools were further stratified based on educational quality. Approximately 30 students from Grade 4, Grade 6, or Grade 8 were then randomly selected from each sampled school. In total, 207 county-level units were randomly selected across 31 provinces, covering seven regions of China: Northeast, North China, East China, Central China, Northwest, Southwest, and South China (Law and Guo, 2022), with 91,762 students from 2,876 schools participating in the study. The survey was administered through a secure online platform with assistance from trained school staff and class teachers. The study strictly adhered to ethical standards, and all participants took part voluntarily and anonymously. Prior to data collection, written informed consent was obtained from participants’ legal guardians or close relatives. All data collection procedures complied with institutional ethical guidelines, ensuring participants’ privacy and minimizing potential risks.

To ensure the quality of the data, we used the Mandata plugin in SPSS 26.0 to examine response consistency. The system automatically detected some questionnaires in which several consecutive items had the same answer, indicating that respondents may not have engaged seriously in answering the questions. Among the 91,762 questionnaires collected, 608 were identified as invalid due to inconsistent responding and were excluded from further analysis. In addition, based on the items measuring the study variables, 3,005 questionnaires with excessive missing data were removed. Ultimately, 88,149 valid questionnaires were retained, producing an effective response rate of 96.06%. The demographic distribution of the sample is presented in Table 1.

**Table 1:** The distribution of control variables (*N* = 88,149).

Variables	Category	Frequency	Percentage
Gender	Male	44,759	50.8%
	Female	43,390	49.2%
School Level	Primary school	68,041	77.2%
	Lower secondary school	20,108	22.8%
School Type	Boarding school	16,813	19.1%
	Non-boarding school	71,337	80.9%

### 3.2 Measures

#### 3.2.1 Assessment of SRL

The SRL questionnaire was developed by authors, with items derived from the Academic Self-Regulated Learning Questionnaire (ASLQ, Nambiar et al., 2022). To ensure the instrument was culturally and linguistically appropriate, we followed a standard translation and back-translation procedure. While the core items remain consistent with the original ASLQ, the phrasing was refined to better suit the Chinese academic environment. The SRL questionnaire consists of 7 items covering two dimensions: the monitoring strategies (3 items) and the cognitive strategies (4 items). The items of monitoring strategies and cognitive strategies were assessed through items such as “I make study plans based on the learning tasks I need to complete each day” and “I use tools such as concept maps, mind maps, and tables to help summarize what I have learned.” The items in this questionnaire are scored on a 5-point Likert scale (1 = “strongly disagree” to 5 = “strongly agree”). A higher total score indicates a higher level of SRL. The results show that the scale exhibits good reliability and acceptable structural validity according to the cut-off values suggested by Hu and Bentler (1999). The model fit indices were as follows: $\chi^{2}$ /df = 310.38 (*p* < 0.001), CFI = 0.995 (> 0.95), TLI = 0.991 (> 0.95), SRMR = 0.014, and RMSEA = 0.059 (< 0.08). In addition, the standardized factor loadings of the seven items ranged from 0.89 to 0.92. The Cronbach’s alpha for the overall questionnaire was 0.97, with values of 0.91 and 0.90 for the two subscales, respectively.

#### 3.2.2 Assessment of academic achievement

Students’ academic achievement was measured through a self-reported item. Prior research has shown that students’ perceptions of their academic performance are highly correlated with their actual grades. This suggests that self-reported GPAs possess high reliability at both the sample and population levels (e.g., Kuncel et al., 2005; Wen et al., 2010; ACT, 2013; Christian et al., 2020). In the current study, students were asked, “Which performance level does your overall academic achievement fall into within your class?” to evaluate their academic achievement across subjects. A five-point scale was used (1 = “Bottom 40% of the class,” 2 = “31%–40% of the class,” 3 = “21%–30% of the class,” 4 = “Top 10%–20% of the class,” 5 = “Top 10% of the class”). Higher scores reflect a higher relative ranking in the class and thus better academic performance.

#### 3.2.3 Assessment of academic emotions

Academic emotions were used as a mediating variable in the current study. The scale was adopted from the Adolescent Academic Emotion Scale (AAEQ, Dong and Yu, 2007), which was originally adapted from Pekrun et al.’ (2002) achievement emotion framework and has been validated within the Chinese cultural context. The scale includes four dimensions: Positive-high arousal (PH-arousal, e.g., pride, happiness, hope; 11 items), positive-low arousal (PL-arousal, 12 items), negative-high arousal (NH-arousal, 13 items), and negative-low arousal (NL-arousal, 22 items). Among them, items such as “I feel proud because I have achieved good grades” reflect the pride dimension of PH-arousal emotions, whereas “I feel anxious before an exam” reflects the anxiety dimension of NH-arousal emotions. All items were rated on a five-point scale (1 = completely inconsistent, 5 = completely consistent). Reliability analysis showed that the internal consistency coefficients for the four subscales were 0.95, 0.97, 0.94, and 0.95, respectively. Additionally, CFA results showed that that the standardized factor loadings ranged from 0.69 to 0.88, 0.76 to 0.87, 0.71 to 0.85, and 0.76 to.88 for the four dimensions. The model fit indices were as follows: $\chi^{2}$ /df = 2118.53, CFI = 0.934, TLI = 0.928, SRMR = 0.058, and RMSEA = 0.078, suggesting that the scale demonstrated acceptable reliability and validity.

#### 3.2.4 Assessment of TSR

The TSR scale used in this study was adapted from the original scale developed by Pianta and further revised by Settanni et al. (2015). To focus on the positive dimension of TSR, seven items reflecting Closeness were selected, consistent with the approach used in previous Chinese studies (Pan, 2015). Sample items include “When I’m with my teacher I feel happy.” All items were rated on a 5-point Likert scale (1 = “strongly disagree,” 5 = “strongly agree”). Cronbach’s alpha for this scale was 0.95, and CFA results showed that the standardized factor loadings of the seven items ranged from 0.85 to 0.88 ( $\chi^{2}$ /df = 2118.53, CFI = 0.951, TLI = 0.926, SRMR = 0.025, and RMSEA = 0.078), suggesting that the scale demonstrated good reliability and acceptable construct validity.

#### 3.2.5 Control variables

To eliminate potential endogeneity issues, this study controlled for key variables influencing students’ academic achievement in the structural equation model. Referring to previous research (e.g., Jiang and Men, 2016), we controlled for variables such as gender (1 = male, 2 = female), educational stage (1 = primary school, 2 = junior high school) and school types (1 = boarding school, 2 = non-boarding school) to avoid potential bias. Furthermore, considering that the level of socioeconomic development may indirectly affect teaching quality and students’ academic achievement, we included seven economic regions in China (e.g., Northeast, North China, East China) as dummy variables in the regression model.

### 3.3 Data analysis

This study used IBM SPSS 22.0 for descriptive statistics, reliability analysis, correlation analysis, and regression analysis. A regression-based path approach was employed, and the PROCESS macro was used to examine the direct effects among SRL, academic emotions, TSR, and academic achievement, as well as the conditional indirect effects by academic emotions. Specifically, Models 4 and 8 of the PROCESS macro were applied. A moderated mediation model was examined within a conditional process framework using the bootstrap procedure. As a non-parametric resampling technique, the bootstrap procedure does not rely on the assumption of a normal distribution, making it particularly robust for testing mediation effects in large-scale data. For all effect analyses, 95% bias-corrected confidence intervals were estimated using 5,000 bootstrap resamples. A *p*-value less than 0.05 was considered statistically significant.

## 4 Results

### 4.1 Common method variance

The data for all variables in the current study were derived from self-reports by the sample students, so there may be a potential common method variance (CMV) issue. To control for the potential CMV, the Harma’s single-factor test was performed before data analysis. This method analyzes whether all variables primarily load onto a single factor, thus reflecting the presence of CMV. According to the results of Harman’ s single-factor test, the eigenvalues of four factors were greater than 1, and the cumulative variance explained by the first factor was 38.8%, which is below 40%. According to Hair et al. (2019), if the variance explained by the first factor is less than 40%, it indicates that CMV does not pose a significant problem. This suggests that no significant CMV was found in this study.

### 4.2 Descriptive analyses

Descriptive statistics and correlation results for all variables are shown in Table 2. Specifically, SRL (*M* = 3.70, SD = 1.01) was significantly positively related to PH-arousal emotions (*M* = 3.80, SD = 0.94; $r_{2}$ = 0.451, p < 0.001), PL-arousal emotions (*M* = 3.48, SD = 1.03; $r_{4}$ = 0.555, p < 0.001), and positive TSR (*M* = 3.89, SD = 1.00; $r_{16}$ = 0.750, p < 0.001). Academic achievement was also significantly positively related with these three variables ( $r_{3}$ = 0.341, p < 0.001; $r_{5}$ = 0.436, p < 0.001; $r_{17}\;$ = 0.288, p < 0.001). In contrast, SRL, positive TSR, and academic achievement were significantly negatively correlated with NH-arousal emotions ( $r_{7}$ = − 0.304, p < 0.001; $r_{20}$ = − 0.318, p < 0.001; $r_{8}$ = − 0.303, p < 0.001) and NL-arousal emotions ( $r_{11}$ = − 0.332, p < 0.001; $r_{12}$ = − 0.323, p < 0.001; $r_{21}$ = − 0.309, p < 0.001).

**Table 2:** Descriptive statistics (*N* = 88,149).

Variables	M	SD	1	2	3	4	5	6	7
1 SRL	3.97	0.95	1						
2Academic achievement	3.70	1.01	0.331^***^	1					
3 PH-arousal	3.80	0.94	0.451^***^	0.341^***^	1				
4 PL-arousal	3.48	1.03	0.555^***^	0.436^***^	0.826^***^	1			
5 NH-arousal	2.33	1.05	−0.304^***^	−0.303^***^	−0.089^***^	−0.279^***^	1		
6 NL-arousal	1.99	1.01	−0.332^***^	−0.323^***^	−0.179^***^	−0.266^***^	0.886^***^	1	
7 Positive TSR	3.89	1.00	0.750^***^	0.288^***^	0.432^***^	0.537^***^	−0.318^***^	−0.309^***^	1

*p < 0.05; **p < 0.01; ***p < 0.001.

### 4.3 Test of the mediating effects

To examine the mediating role of academic emotions in the relationship between SRL and academic achievement, the model incorporates four dimensions of academic emotions: PH-arousal, PL- arousal, NH-arousal, and NL-arousal. Referring to the previous study (Hayes, 2013), we use the model 4 of PROCESS plugin in SPSS 22.0 to examine the mediation model. The results are shown in Tables 3, 4. After controlling for covariates, results showed that, when the mediator was not included, SRL significantly was correction with academic achievement ( β = 0.336, *t* = 99.231, p < 0.001). Specifically, the total effect of SRL on academic achievement was 0.336, with a 95% confidence interval of [0.329, 0.343].

**Table 3:** The regression results of mediation model.

Dependent variable	Independent variables	$R^{2}$	F	β	t
Academic achievement	Gender	0.122	2439.755^***^	0.09	14.170^***^
	Educational stages			−0.215	−27.205^***^
	School type			0.009	1.011
	Region			−0.024	−13.574^***^
	SRL			0.316	99.231^***^
PH-arousal	Gender	0.208	4618.697^***^	0.03	5.265^***^
	Educational stages			0.019	2.653**
	School type			0.106	14.034^***^
	Region			−0.022	−12.755^***^
	SRL			0.44	147.521^***^
PL-arousal	Gender	0.314	807.780^***^	−0.027	−1.644^***^
	Educational stages			−0.119	−16.566^***^
	School type			0.024	3.081**
	Region			−0.038	−19.514^***^
	SRL			0.59	193.765^***^
NH-arousal	Gender	0.103	2015.085^***^	0.067	9.947^***^
	Educational stages			0.137	16.384^***^
	School type			−0.04	−4.488^***^
	Region			0.045	21.799^***^
	SRL			−0.323	−91.141^***^
NL-arousal	Gender	0.117	2341.248^***^	−0.013	−2.102*
	Educational stages			0.136	17.025^***^
	School type			−0.078	−9.097^***^
	Region			0.026	13.220^***^
	SRL			−0.339	−1.286^***^
Academic achievement	Gender	0.246	3196.853^***^	0.098	16.503^***^
	Educational stages			−0.147	−19.717^***^
	School type			−0.013	−1.595^***^
	Region			−0.009	−4.773^***^
	SRL			0.072	18.712^***^
	PH-arousal			−0.032	−5.097^***^
	PL-arousal			0.335	58.997^***^
	NH-arousal			0.001	0.172
	NL-arousal			−0.204	−29.853^***^

*p < 0.05; **p < 0.01; ***p < 0.001.

**Table 4:** Direct and indirect effects of SRL on students’ academic achievement.

Path	Effect	Proportion of total effect	95% CI	
			Boot LLCI	Boot ULCI
SRL → Academic achievement (direct effect)	0.072	21.428%	0.064	0.080
SRL → PH-arousal → Academic achievement	−0.014	4.166%	−0.020	−0.008
SRL → PL-arousal → Academic achievement	0.209	62.202%	0.201	0.217
SRL → NL-arousal → Academic achievement	0.069	2.535%	0.064	0.074
SRL → Academic achievement (total effect)	0.336		0.329	0.340

When academic emotions were added as a mediator, SRL was positively associated with PH-arousal ( β = 0.440, *t* = 147.521, p < 0.001) and PL-arousal ( β = 0.590, *t* = 193.765, p < 0.001), while negatively associated with NH-arousal ( β = − 0.323, *t* = 91.141, p < 0.001) and NL-arousal ( β = − 0.339, *t* = −100.28, p < 0.001). When the mediator was added to the model, the direct effect of SRL on academic achievement decreased to 0.072, with a 95% confidence interval of [0.064, 0.080], because the interval did not include 0, the direct effect remained significant ( β = 0.072, *t* = 18.712, p < 0.001).

Moreover, PL-arousal was positively correlated with academic achievement ( β = 0.335, *t* = 58.997, *p* < 0.001), whereas PH-arousal was negatively correlated with academic achievement ( β = − 0.032, *t* = −5.097, p < 0.001). The mediating effects of PH-arousal and PL-arousal accounted for 4.2 and 62.2% of the total effect, respectively. The mediating effects of PH-arousal and PL-arousal were −0.014 and 0.209, with 95% confidence intervals of [−0.020, −0.008] and [0.201, 0.217], respectively, which did not include 0, reflecting significant mediating effects.

On the contrary, NL-arousal was negatively associated with academic achievement ( β = − 0.204, *t* = −29.853, p < 0.001), with the mediating effect of NL-arousal being 0.069 and a 95% confidence interval of [0.064, 0.074], which did not include 0, accounting for 20.2% of the total effect. The effect of NH-arousal was not significant (*β* = 0.001, *t* = 0.172, p > 0.05).

### 4.4 Test of moderated mediation

Given that the theoretical assumptions underlying this study posit that the moderator only affects the direct path and the first stage of the mediation process, we did not examine its potential effects on the second stage of the mediation pathway. Therefore, the interaction terms U × W (i.e., b1, b2, b3, b4) were not included. Accordingly, PROCESS Model 8 was applied to test the moderated mediation model of TSR. Then, a bias-corrected percentile bootstrap method was applied to assess the significance of the moderating effects. The results are presented in Table 5. As shown in Table 5, SRL shows a positive relationship with students’ academic achievement, and the interaction term was significant (*β* = 0.022, *t* = 2.096, p < 0.001), with a confidence interval of [0.002, 0.043], which does not include zero. The interaction between SRL and positive TSR was also significantly associated with PH-arousal emotions (*β* = 0.030, *t* = 12.312, p < 0.001) and PL-arousal emotions (*β* = 0.102, *t* = 45.516, p < 0.001), with confidence intervals of [0.025, 0.035] and [0.105, 0.115], respectively, neither of which includes 0. In addition, the interaction between SRL and TSR was significantly associated with academic achievement (*β* = 0.021, *t* = 8.514, p < 0.001). These results indicate that TSR moderates both the direct effect and the first stage of the mediation process.

**Table 5:** The conditional mediation effects of positive TSR on Students’ academic achievement.

Path	β	t	95% CI	
			Boot LLCI	Boot ULCI
SRL → Academic achievement	0.022	2.096^*^	0.002	0.043
SRL × Positive TSR → Academic achievement	0.021	8.514^***^	0.016	0.026
SRL × Positive TSR → PH-arousal	0.030	12.312^***^	0.025	0.035
SRL × Positive TSR → PL-arousal	0.102	45.516^***^	0.097	0.106
SRL × Positive TSR → NL-arousal	−0.069	−26.528^***^	−0.074	−0.064

*p < 0.05; **p < 0.01; ***p < 0.001.

Table 6 shows the mediating effects of PH-arousal, PL-arousal, and NL-arousal emotions on academic achievement, and it provides the corresponding 95% bootstrap confidence intervals for the three levels of positive TSR (M - 1 SD, M, and M + 1 SD).

**Table 6:** Mediating effects of academic emotions across different levels of positive TSR.

Mediator	Positive TSR	Conditional indirect effect	Boot SE	95% CI	
				Boot LLCI	Boot LLCI
PH-arousal	2.89 (M-1SD)	−0.007	0.002	−0.010	−0.004
	3.89 (M)	−0.008	0.002	−0.011	−0.004
	4.89 (M + 1SD)	−0.008	0.002	−0.012	−0.004
PL-arousal	2.89 (M-1SD)	0.105	0.003	0.100	0.110
	3.89 (M)	0.142	0.003	0.135	0.148
	4.89 (M + 1SD)	0.178	0.004	0.170	0.186
NL-arousal	2.89 (M-1SD)	0.039	0.002	0.035	0.042
	3.89 (M)	0.053	0.002	0.049	0.057
	4.89 (M + 1SD)	0.067	0.003	0.062	0.072

**Figure 2:**
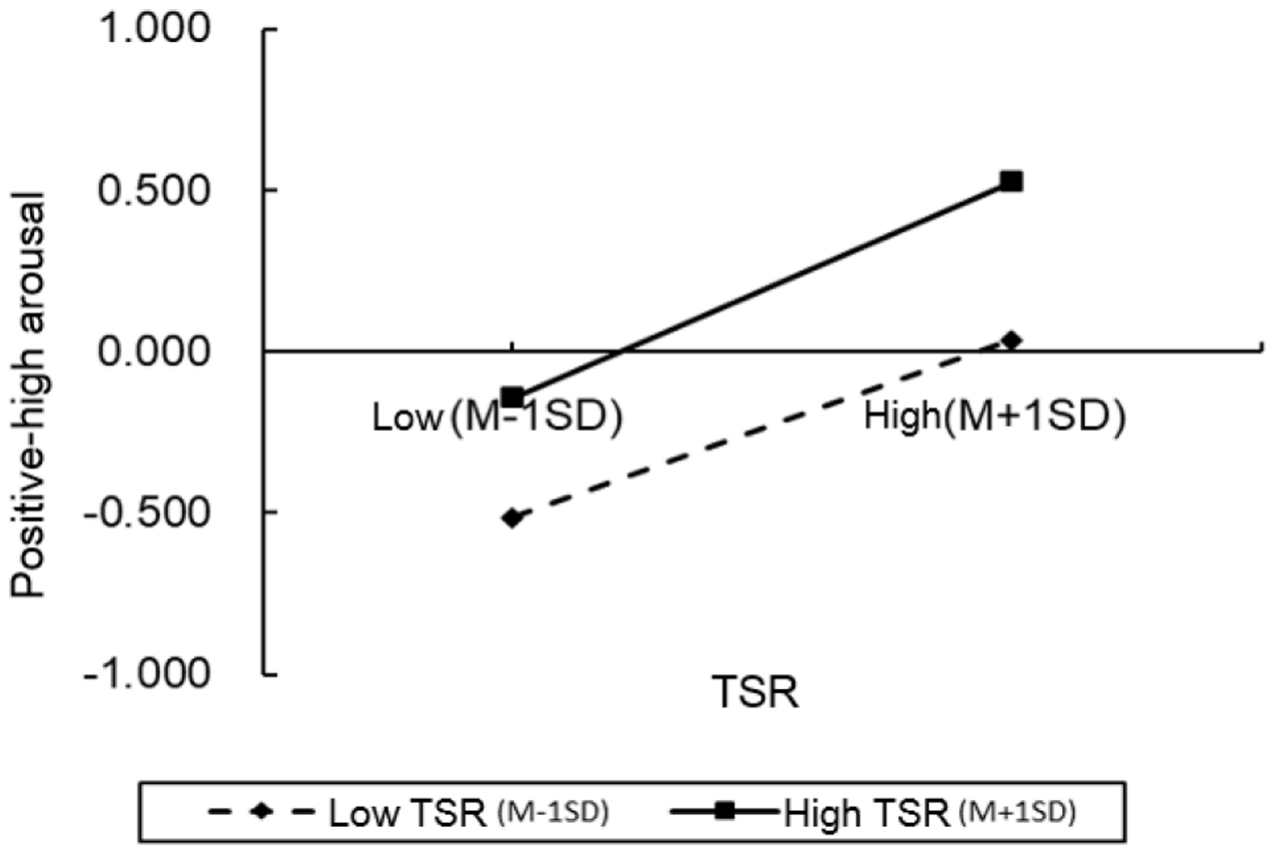
The moderating effects of positive TSR on PH-arousal.

**Figure 3:**
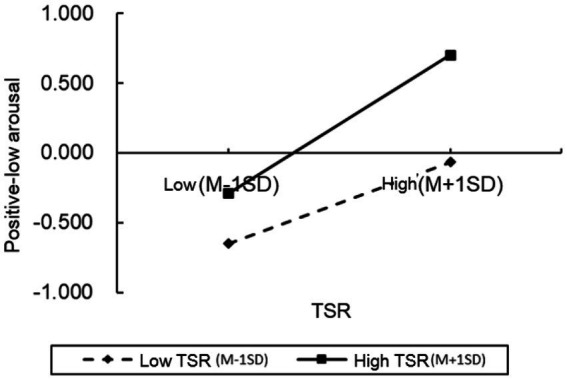
The moderating effects of positive TSR on PL-arousal.

**Figure 4:**
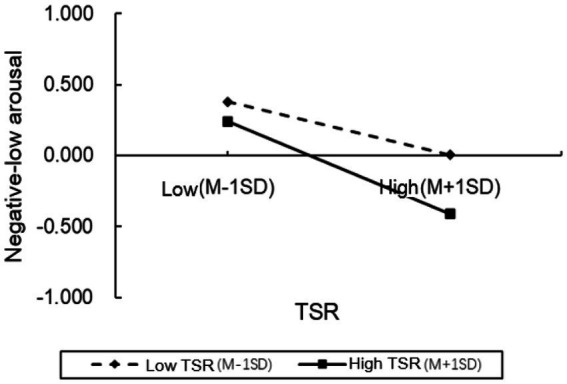
The moderating effects of positive TSR on NL-arousal.

**Figure 5:**
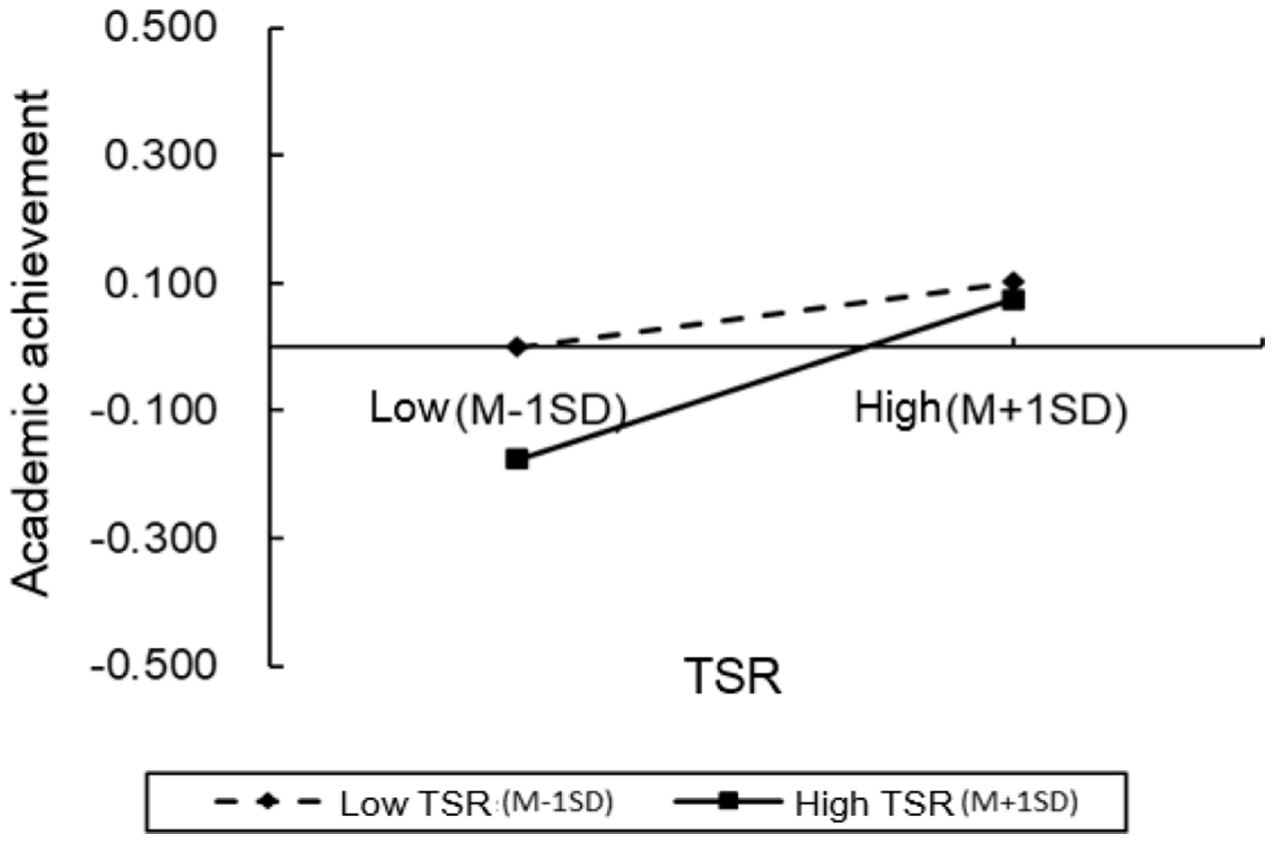
The moderating effects of positive TSR on student’s academic achievement.

To further examine the moderation effect in greater detail, we conducted simple slope analyses and plotted simple effect graphs (see Figures 2–5). The results presented that when the level of positive TSR was low (M − 1 SD), SRL was positively associated with PH-arousal emotions ( $\beta_{\text{simple}}$ = 0.274, p < 0.001), PL-arousal emotions ( $\beta_{\text{simple}}$ = 0.293, p < 0.001), and academic achievement ( $\beta_{\text{simple}}$ = 0.083, p < 0.001), while being negatively associated with NL-arousal emotions ( $\beta_{\text{simple}}$ = − 0.326, p < 0.001). When positive TSR was high (M + 1 SD), the positive association of SRL with PH-arousal emotions became stronger ( $\beta_{\text{simple}}$ = 0.334 vs. 0.274, *p* < 0.001), with PL-arousal emotions ( $\beta_{\text{simple}}$ = 0.496 vs. 0.293, p < 0.001), and with academic achievement ( $\beta_{\text{simple}}$ = 0.125, p < 0.001). Similarly, higher levels of positive TSR attenuated NL-arousal emotions and strengthened the negative association between SRL and NL-arousal emotions ( $\beta_{\text{simple}}$ = − 0.326 vs. −0.188, p < 0.001). These findings indicate that as the level of positive TSR increases, the positive association between SRL and students’ positive academic emotions (PH-arousal and PL-arousal) becomes stronger, while its association with negative academic emotions (NL-arousal) is weakened. In other words, students tend to report higher levels of positive academic emotions during SRL when positive TSR are higher.

## 5 Discussion

This study examined the relationship between SRL and academic achievement, as well as the underlying mechanisms driving this association. The results showed that SRL was indirectly associated with academic achievement via academic emotions, and that this indirect association, along with the direct path between SRL and academic achievement, was moderated by positive TSR. The results provide empirical support for the dual-processing model of students’ learning behaviors and academic emotions. These two processes are theoretically interrelated, consistent with models suggesting that students’ goal-oriented engagement and evaluation during task interactions facilitate the generation of academic emotions (Feraco et al., 2023; Pekrun, 2006). Overall, these results offer empirical evidence and practical insights for promoting college students’ SRL and the regulation of their academic emotions.

### 5.1 SRL and academic achievement among adolescent

The impact of SRL on academic achievement is a complex and dynamic process, involving a cyclical relationship among the forethought, performance, and reflection phases (Caixia et al., 2025). According to SRLT, students with higher levels of SRL are better able to manage their learning processes, make more rational decisions during learning tasks, and strategically select and optimize learning strategies, thereby enhancing their academic performance. This conclusion has been consistently supported by a substantial body of empirical research (e.g., Pylväs et al., 2022; Liang et al., 2023). Consistent with these findings, the present study demonstrates that higher levels of SRL are associated with higher academic achievement, further supporting the core assumptions of SRLT.

Moreover, the 2018 PISA report identified autonomous learning as one of the key competencies required for students in the 21st century, underscoring its critical role in promoting both academic success and social adaptability (OECD, 2019). Prior research has shown that in remote and blended learning environments, students face heightened demands for autonomy, thereby amplifying the influence of SRL on academic achievement (Lobos et al., 2024). In the contemporary era of rapid advances in artificial intelligence, although intelligent technologies offer substantial convenience and personalized support for learning, they may also, to some extent, undermine students’ capacities for active thinking, sustained engagement, and self-monitoring (Lan and Zhou, 2025). Meanwhile, influenced by Confucian cultural traditions and related contextual factors, Asian students generally exhibit lower levels of learning autonomy, with Chinese adolescents’ autonomous learning abilities remaining at a low to moderate level (e.g., Bai et al., 2025). Taken together, these findings highlight the urgency and practical significance of systematically fostering and strengthening adolescents’ SRL level in the AI era.

### 5.2 The mediating effect of academic emotions

This study found that SRL is a positively associated with positive academic emotions and negatively associated with negative academic emotions. This finding confirms the cyclical relationship between SRL and emotions. Individuals with an autonomous motivation orientation exhibit higher levels of self-regulation, cognitive flexibility, and positive emotions, while positive academic emotions further facilitate the optimization of learning strategies (Brenner, 2022). Moreover, research by Zhao et al. (2024) confirmed that students’ ability to use effective technology-assisted learning in smart classrooms tends to be linked to higher levels of positive academic emotions. Therefore, the findings of this study align with previous research and provide China-specific evidence supporting the “cognition–academic emotion” cyclical model.

In addition, this study found that four types of academic emotions—PH-arousal, PL-arousal, NH-arousal, and NL-arousal—significantly affect academic achievement. Academic emotions play a mediating role in the path through the association between SRL levels and students’ academic performance. The Assessment-Learning Integration model emphasizes promoting learners’ cognitive development and the effective use of cognitive strategies through coordinated peer and self-assessment, thereby stimulating students’ interest and confidence in self-regulated learning (Xin et al., 2025; Zhang et al., 2023). Task-oriented and inquiry-based learning serve as pathways for students’ SRL, helping them gain a sense of accomplishment through hands-on practice and engagement, which in turn enhances academic performance (Bransford et al., 1999). Thus, leveraging the interactive effect of SRL and academic emotions is crucial for improving academic achievement.

### 5.3 The moderating effect of positive TSR

This study further demonstrates that positive TSR moderates both the first half of the “SRL→ academic emotions → academic achievement” pathway and the direct “SRL → academic achievement” pathway. In addition, positive TSR also moderates the direct effect of SRL strategies on academic achievement. Specifically, under the same level of SRL, students with higher levels of positive TSR exhibit higher levels of positive academic emotions compared with those with lower levels. Previous research has shown that students who develop positive relationships with their teachers are more likely to experience positive emotions such as relaxation, enthusiasm, inspiration, and higher learning satisfaction, which in turn enhances academic achievement (e.g., Li and Zhang, 2024; Carmona-Halty et al., 2024).

Moreover, studies indicate that when learning tasks are cognitively demanding, the use of SRL strategies may actually increase students’ cognitive load and elicit higher levels of stress and anxiety (Marsch et al., 2024). Likewise, insufficient social support and excessive external pressures may negatively impact the relationship between SRL and academic emotions, undermining the beneficial effects of self-regulation on learning outcomes (Nabizadeh et al., 2019). However, emotion regulation skills help students maintain emotional stability in high-pressure learning environments, thereby alleviating anxiety, improving attention, and enhancing academic performance (Kassab et al., 2015; Ghanizadeh, 2017). In this process, positive TSR is considered a key contextual resource that facilitates students’ emotion regulation during self-regulated learning (Dan and Bai, 2025). Accordingly, supportive TSR provides a stable emotional foundation for learning, enabling students to maintain positive emotions during learning or preserve emotional stability when facing learning challenges and cognitive load, which in turn supports more effective regulation of learning engagement and behaviors (La Paro et al., 2004). These findings further reinforce the empirical support for attachment theory and guided educational dialog theory.

Furthermore, this study found that positive TSR significantly moderates the direct pathway from self-directed learning ability to academic achievement, which is consistent with prior research (e.g., Mauring and Acledan, 2025). Teachers with a growth mindset tend to interact with students in a more egalitarian manner. Research has shown that such teachers respond constructively to challenges and mistakes, which in turn enhances students’ resilience when facing setbacks and improves their self-directed learning abilities. This provides further evidence for the crucial role teachers play in promoting students’ self-directed learning development (Bai et al., 2025; Lou and Noels, 2019).

## 6 Implications and limitations

### 6.1 Theoretical implications

This study draws on Pintrich’s self-regulated learning (SRL) model and employs a moderated mediation framework to examine the relationships among adolescents’ self-regulated learning, academic emotions, positive TSR, and academic achievement. The main theoretical implications of this study are as follows:

First, this study provides large-scale empirical evidence that is consistent with the cyclical and recursive assumptions of Pintrich’s SRL model. The findings indicate that SRL is not only directly associated with students’ academic achievement but also shows an indirect association with their performance via different types of academic emotions (Pintrich, 2000, 2003). This pattern validates an alternative internal logic underlying the relationship between SRL and academic emotions suggested by previous research, and it also supports the learning–assessment integration perspective, which posits that peer assessment enhances students’ effective use of strategies and strengthens their learning confidence (Zhang et al., 2023). In addition, as an important contextual factor, teacher–student relationships shape the emotional experiences adolescents undergo during the SRL process, which in turn further influence their academic achievement. These empirical findings not only enrich and extend the theoretical framework of Pintrich’s model but also enhance our understanding of the mechanisms of self-regulated learning in upcoming digital learning environments.

Second, this study provides empirical support for the TSR role conceptualized in attachment theory (Ainsworth et al., 2015). According to attachment theory, the sense of security that individuals derive from significant others is a core foundation for emotional regulation, exploratory behavior, and adaptive development. In the academic context, teachers function as “substitute attachment figures” for students, providing emotional security that facilitates the experience of positive academic emotions (Lei et al., 2018; Wang et al., 2024). This study demonstrates that TSR constitute an important environmental factor influencing students’ emotions, behaviors, and academic development. Specifically, positive TSR can enhance students’ positive academic emotions while reducing negative academic emotions.

Furthermore, the interaction between SRL and positive TSR positively moderates students’ academic emotions, thereby promoting improvements in academic achievement. This finding is consistent with the view that close TSR can positively predict students’ learning interest, learning confidence, and other motivational factors, which in turn enhance academic achievement. These results suggest that during the process of SRL, appropriate and supportive teacher–student interactions can improve students’ positive psychological motivation and encourage them to use more flexible and effective learning strategies, ultimately improving academic performance.

### 6.2 Practical implications

Based on the research findings, this study offers practical recommendations to foster the positive academic achievement associated with students’ SRL and to improve students’ SRL skills, particularly in the following three key aspects.

First, the findings of this study demonstrate that self-regulated learning (SRL) is significantly and positively associated with positive achievement emotions, while being negatively associated with negative achievement emotions. Consequently, pedagogical practices should focus on enhancing students’ autonomous learning capacities while maintaining a high sensitivity to fluctuations in their emotional states. Given the pivotal mediating role of achievement emotions identified in our model, educators should implement targeted intervention measures, such as establishing emotional monitoring and support mechanisms. These can help students utilize cognitive reappraisal strategies to maintain a positive mindset and alleviate negative arousal during the self-regulation process, thereby reducing cognitive load and psychological strain. Furthermore, the path associations between SRL and achievement emotions suggest a reciprocal, ‘cyclical’ structure: students with higher SRL proficiency tend to possess a stronger sense of mastery and self-efficacy, which in turn elicits positive affect; conversely, positive emotions drive students to further refine and optimize their SRL strategies.

Second, TSR exhibited a moderating role, not only influencing the first stage of the mediation path (SRL → academic emotions → achievement) but also bolstering the direct association between SRL and academic achievement. Therefore, teachers should leverage the synergistic effects between self-regulated learning and academic emotions, and strengthen interpersonal relationships and emotional support to stimulate students’ intrinsic motivation. Furthermore, educators should encourage multi-stakeholder participation in the deep integration of “assessment and learning”. This involves utilizing self-reflection as a core regulatory mechanism, complemented by peer assessment as an external motivational tool, enabling students to gain a sense of accomplishment and enhance their self-efficacy through the evaluation process.

Third, professional teacher training should focus on improving educators’ sensitivity and discernment regarding the diversity of academic emotions, with a particular emphasis on accurately identification positive high-arousal (PH-arousal) and negative low-arousal (NL-arousal) states. By providing personalized emotional regulation guidance, teachers can effectively mitigate learning fatigue and frustration, thereby supporting students in maintaining a balanced and relaxed mindset to optimize learning outcomes.

### 6.3 Research limitations

There are some limitations in this study. First, the use of cross-sectional data limits our ability to determine the temporal ordering and causal relationships among variables. For instance, relying solely on structural equation modeling to examine the relationship between SRL and academic emotions supports Pintrich’s theoretical assumption of a potential cyclical model, but it does not allow for the identification of actual recursive processes over time. Therefore, future research should employ experimental or longitudinal designs to more clearly investigate temporal sequences and potential causal relationships.

Second, this study assessed students’ academic achievement solely through self-reported measures. Although numerous studies have shown that self-reported grades are significantly correlated with objective academic performance, this measurement approach remains subjective and may be affected by reference bias. Therefore, the relationships among variables and the strength of these associations in the present study were interpreted with caution. Future research could consider using standardized test scores or other objective academic indicators to conduct more comprehensive and precise analyses.

Third, this study examined only academic emotions and TSR. The association between SRL and academic achievement may also vary depending on other factors involved in the self-regulation process, such as time management or motivational levels. Future research may thus further explore these additional factors and their roles within the broader mechanism.

## 7 Conclusion

The main conclusions of this study are as follows: (1) SRL is positively associated with academic achievement. (2) PH-arousal, PL-arousal, and NL-arousal mediated the connection between SRL and academic achievement, while NH-arousal did not. (3) TSR significantly moderates the direct pathway of SRL on academic achievement. (4) Positive TSR also significantly moderates the associations between SRL and three types of academic emotions which mentioned above.

## Data Availability

The raw data supporting the conclusions of this article will be made available by the authors, without undue reservation.
