# Correction: The relationship between adolescents' self-regulated learning and academic achievement: an interrelated mediation model of academic emotions: evidence from a nationwide sample in China

**DOI:** 10.3389/fpsyg.2026.1828947

**Published:** 2026-03-27

**Authors:** Rui Sun, Sheng Zhang

**Affiliations:** Collaborative Innovation Center of Assessment toward Basic Education Quality, Beijing Normal University, Beijing, China

**Keywords:** a large survey, academic achievement, academic emotions, self-regulated learning, teacher-student relationships

The original article erroneously stated that financial support was not received for the work and/or its publication. The correct **Funding** appears below:

The author(s) declared that financial support was received for this work and/or its publication. This research was supported by the Chinese Society of Education 2022 Key Planned Education Research Project, “Research on the Evaluation Framework and Practical Effects of After-School Services in Primary and Secondary Schools” (Grant No. 202200042101A).

The original version of this article has been updated.

